# Competition and habitat availability interact to structure arboreal ant communities across scales of ecological organization

**DOI:** 10.1098/rspb.2023.1290

**Published:** 2023-09-27

**Authors:** Benjamin J. Adams, Evan M. Gora, Matina C. Donaldson-Matasci, Elva J. H. Robinson, Scott Powell

**Affiliations:** ^1^ Department of Biological Sciences, George Washington University, Washington, DC, USA; ^2^ Smithsonian Tropical Research Institute, Balboa, Panama; ^3^ Cary Institute of Ecosystem Studies, Millbrook, NY, USA; ^4^ Department of Biology, Harvey Mudd College, Claremont, CA 91711, USA; ^5^ Department of Biology, University of York, York, UK

**Keywords:** community ecology, habitat filtering, competition, resource limitation, species coexistence

## Abstract

Understanding how resource limitation and biotic interactions interact across spatial scales is fundamental to explaining the structure of ecological communities. However, empirical studies addressing this issue are often hindered by logistical constraints, especially at local scales. Here, we use a highly tractable arboreal ant study system to explore the interactive effects of resource availability and competition on community structure across three local scales: an individual tree, the nest network created by each colony and the individual ant nest. On individual trees, the ant assemblages are primarily shaped by availability of dead wood, a critical nesting resource. The nest networks within a tree are constrained by the availability of nesting resources but also influenced by the co-occurring species. Within individual nests, the distribution of adult ants is only affected by distance to interspecific competitors. These findings demonstrate that resource limitation exerts the strongest effects on diversity at higher levels of local ecological organization, transitioning to a stronger effect of species interactions at finer scales. Collectively, these results highlight that the process exerting the strongest influence on community structure is highly dependent on the scale at which we examine the community, with shifts occurring even across fine-grained local scales.

## 1. Introduction

Resource-mediated habitat filtering and competition are expected to interact in the structuring of ecological communities [1–3]. The resources available in a habitat set the foundation for which species can live there, based on the fundamental niche and minimum resources required for long-term persistence of each species [4,5]. For each species that finds suitable resources, their persistence in the community can then be challenged by competition, potentially leading to exclusion [6–8]. Considerable work has focused on detecting the signatures of habitat filtering, competition and their interactions at large spatial scales [9,10]. Such work summarizes broad hierarchical patterns across a diversity of habitats, with habitat filtering generally being more important at large scales but with the balance shifting to competition at finer scales. However, the direct mechanisms underlying patterns of diversity are frequently obscured at broader spatial scales [11,12]. At the other extreme, local spatial scales provide opportunities to directly link known resource usage and competitive interactions among individuals with observable community patterns [13,14]. Nevertheless, studies that address the relative importance of resource availability and competition in structuring communities across multiple local scales are rare (but see work with communities in pitcher plants [15] and rock pools [16]). Filling this knowledge gap is critical for understanding the extent to which local processes and their interactions scale to influence the structure of ecological communities [14,17].

At local spatial scales, the influences of resource availability and biotic interactions on community structure are thought to be spatially dependent and complex [14]. Available resources often occur in discrete patches that limit access for species in the community, especially if dispersal to new resource patches is challenging [18,19]. Although frequently simplified to pairwise, linear interactions between species [20], communities generally exist as an assemblage of multiple species differentially interacting across space [21,22]. Indeed, how species use and partition resources in space is often complex and variable at local scales, but one common and understudied context is when individuals acquire resources and grow via establishing and expanding networked patches or nodes of resources. Examples of network patterns of resource acquisition and use span such disparate systems as fungal hyphae and root networks [23] and the foraging and multi-nest networks of social insect colonies [24–26]. Competition in these cases then plays out via interactions between abutting or intertwined networks, with any pattern of network growth facing an array of competitive pressures from multiple species and locations simultaneously. How resource distribution, network growth and competitive interactions shape local-scale patterns of coexistence should thus be highly dependent on fine-scale spatial relationships. Systems that incorporate these layers of local community interactions should then be ideal for addressing the broader knowledge gap of how local processes scale to community-level influences.

Arboreal ant communities are particularly tractable systems for studying the interactions between resource availability and competition in the context of growth via discrete expansion of resource networks [27]. Individual trees function as the primary habitat patches for arboreal ants to colonize and compete over. This is true even within dense forest environments, due to the phenomena of crown shyness and because arboreal ants rarely leave the crowns of trees [28,29]. As a result, arboreal ant communities within an individual tree typically act as isolated ‘island-like’ communities following classic species–area relationships [30–32]. Within a tree, resident colonies of ants also compete fiercely for a limited number of pre-existing nesting cavities [33–36]. Each colony must acquire and defend multiple nest sites, without the capacity to make more themselves, in order to grow and successfully reproduce [37,38], building a nest network as they do so. Finally, individual nests, which represent the finest spatial scale, vary in properties of quality [39] and defensibility [38,40], as well as in the competitive pressures they face [41]. Colonies must make collective decisions about which individual cavities they use within the resource-limited and highly competitive environment they occupy, and how they allocate colony members to a nest to maximize overall colony growth and reproduction [38,39,41].

Here, we use an arboreal ant study system, where growth is via network expansion, to explore the interactive effects of resource availability and competition on community structure at multiple spatial scales. More specifically, we focus on the following three local scales: (i) a discrete resource patch, represented by the whole tree, (ii) resource networks built by organisms, represented by the within-tree nest networks of the resident ant colonies, and (iii) an individual resource within a network, represented by the individual nest. Our central hypothesis is that resource availability has an overarching influence on local community structure, and that species interactions emerge as more important at finer-grained local scales. We tested this hypothesis by surveying arboreal ant communities, quantifying nest site availability, mapping the spatial distribution of nests and quantifying the contents of individual nests. At the patch scale, we evaluated how the availability of resources and the competitive context on whole trees influences the ant community. At the scale of the resource network, we mapped nest networks and evaluated how these are shaped by tree characteristics, ant species and competitive context. Finally, at the scale of an individual resource or node within a resource network, we examined how competition and habitat limitations influenced the populations of adult ants and brood within individual nests. We expect that resource availability will be more important than competition for determining ant community metrics, such as species richness and nest abundance, measured at the scale of an individual tree. By contrast, we expect competition to determine nest selection and ant distribution, with less competitive ant species selecting nest sites and distributing adult ants and brood further away from competitors. Taken together, these detailed data across multiple spatial scales provide an integrative approach to identifying how local resource availability and competition shape community structure, including richness, composition and physical location within a habitat.

## 2. Methods

### (a) Study site and focal species

We conducted all field work at the Dagny Johnson Key Largo Hammock Botanical State Park in Key Largo, Florida, USA (25.178°N, 80.366°W; hereafter Dagny State Park). The Florida Keys are marked by a mild subtropical climate with mean monthly temperatures ranging between 17.9°C and 31.9°C with approximately 101.2 cm of precipitation annually (https://climatecenter.fsu.edu/products-services/data/1981-2010-normals/key-west). Dagny State Park was established in 1982 and hosts the largest remnant in the United States of West Indian hardwood hammock forest [42–44].

The hardwood hammock forest of the Florida Keys present a novel opportunity to work in an arboreal ant system that overcomes many of the logistical constraints of other arboreal ant communities. For example, in the highly diverse tropical forest habitats where most arboreal ant research has focused, felling trees [45] or specialized equipment and training [46–48] are required simply to access the nesting ecology of the ants. Across all tropical habitats, including those that are more accessible, community diversity of more than a hundred species and tree-level diversity of 20 species or more [30,32,40,49] remains a challenge for understanding detailed species interaction or resource requirement. By contrast, the hardwood hammock forest of the Florida Keys is a species-rich ecosystem of conservation concern [43] that has a low and easily accessible canopy (does not exceed 10 m and is frequently less than 6 m; electronic supplementary material, figure S1) [42]. While the arboreal ant diversity is reduced compared with tropical habitats, tropical arboreal ant genera are still well-represented in the full community [50], and all species rely on the same nesting resource for growth and reproduction: hollow cavities in dead stems that are often the abandoned feeding tunnels of wood-boring beetles [51]. Capturing all fine-grained local-scale interactions for even this reduced community of Florida Keys hammock forest could be overwhelming logistically, but most ant communities are dominated by a smaller subset of especially abundant species that capture an array of competitive interactions. Our surveys revealed four especially common species in the hammock forest system that will be our focus here. In addition to high colony incidence across surveyed trees, including frequent co-occurrence (below), these four species also represented contrasting ecology within the larger community. Thus, while this study is not an exhaustive study of the full arboreal ant community in the system, it uses the most abundant players in the community that span an array of potentially generalizable interactions and outcomes. Our four focal species are as follows: (i) *Pseudomyrmex ejectus* and (ii) *Pseudomyrmex simplex*, which are established native species that share similar niche space [52–54], (iii) *Pseudomyrmex gracilis*, which is a disruptive non-native with invasive potential [55,56] and (iv) *Cephalotes varians*, a native species with known defence specialization in its use of nesting resources [57–59].

### (b) Identifying ant colonies and nest locations

We used a combination of multiple baiting and hand-collecting methods [38,60] to document ants foraging and nesting on 176 individual poisonwood trees (*Metopium toxiferum*) in relatively open areas of the hammock forest, in which individual trees are typically physically isolated from other trees. We specifically targeted trees that were not embedded in the larger forest canopy to eliminate any potential connectivity between neighbouring crowns and ensure that the only ants foraging at baits were nesting within the tree [32,61]. Baits (a combination of approx. 140 g of canned chicken and approx. 60 ml of honey with urine added as an additional attractant for *C. varians*) were placed throughout the entire crown of each tree at 12.00 and were examined and refreshed until 21.00 in order to document activity of both the diurnal (*P. ejectus*, *P. gracilis* and *P. simplex*) and nocturnal species (*C. varians*). We selected a subset of the trees (*n* = 31) based on ease of access to the entire crown of the tree and a stratified sampling of tree sizes, and we then tracked foraging ants back to their nests. This method was used to locate all nests of all four of our focal species on each tree and to look for aggressive interactions among conspecifics at baits to ensure all conspecific ants within a tree were from the same colony [38]. Voucher specimens of all ants found at baits or on any other part of the tree were collected and stored in 95% ethanol to be identified in the lab using keys and voucher specimens [62]. It is worthwhile to note that, within the genera of interest, only our focal species of *Cephalotes* is found in the system, and that only our three focal species of the genus *Pseudomyrmex* were found on our study trees in the hammock forest, even though other members of the genus are found in the Florida Keys more generally [50].

### (c) Measurements across local spatial scales

#### (i) Resource patch scale: whole tree

For each tree included in the initial survey, GPS coordinates were recorded, and the diameter of the trunk at 10 cm above the ground was measured. Although diameter at breast height (1.3 m above the ground) is a more common measure, nearly all of the trees in this forest branch below breast height (average distance to first branch = 83.6 cm; range = 10–173 cm in the present study). We used the diameter measured at 10 cm to calculate basal area (BA = *π*(*D*/2)^2^) as a proxy for tree size and an estimate of the total resource patch size for the local ant community [30,63].

For each tree, we also quantified the total amount of dead wood (i.e. the nest resources available to the ant community) in the crown using three methods that required increasing degrees of time and effort in the field but provide increasing resolution of the total resource availability. First, using visual surveys conducted by at least two individuals, we estimated the total per cent dieback for each tree crown to the nearest 5% and took the average between the two when different [64]. We multiplied this percentage by the basal area to produce a weighted proxy of available resources that accounted only for dead wood (i.e. the actual nesting resource). We also counted the total number of dead stems present in each tree crown. Finally, we quantified the total volume of dead wood in each tree by measuring every piece of dead wood by hand and calculating individual stem volume using Newton's Formula (L (A_base_ + 4A_middle_ + A_tip_)/6) which was then summed for the whole tree [65].

#### (ii) Resource network scale: nest network

In ant nest networks, reducing the number of nodes or junctions that an individual ant has to traverse may be more important for travel time than reducing physical distances between nests [24,66,67]. Therefore, we measured all possible paths between every nest and all other nests in a tree, recording all intervening junctions between nest pairs (e.g. branching forks in a tree stem, vines crossing a tree stem, two stems crossing each other or leaves from one stem touching another branch) to generate two distance measurements: the shortest physical distance between nest pairs and the smallest number of junctions between nest pairs. Physical distance was measured as the minimum distance an ant needs to walk between two nests (in cm) and ‘junction distance’ was measured as the fewest number of junctions encountered along any path between two nests. These measurements each produce two types of nest networks: (i) a community-wide nest network for a tree that includes all ant nests and (ii) an intraspecific nest network for each resident ant species that connects only nests within a colony together.

#### (iii) Individual resource scale: individual nests

For every stem containing an ant nest, we calculated stem volume using Newton's Formula as described above and then destructively harvested the stem at the end of the study. To harvest, we visited each nest at a time of day when each target species was not active, sealed all nest entrances and then removed the entire stem from the tree. This ensured all colony members were in their respective nests at the time of collection, and none escaped subsequently. Collected nests were shipped overnight back to the lab where they were immediately frozen at −20°C. All nests were dissected in the lab and the contents were quantified. Specifically, for each nest we confirmed the identity of the resident species and counted all eggs, larvae, worker pupae, soldier pupae, queen pupae, workers, soldiers, alate queens, dealate queens and males.

### (d) Statistical analyses

#### (i) Whole tree analyses

We tested how tree characteristics shaped the arboreal ant community at the scale of an individual tree. We used linear regression to explore whether ant species richness or total ant nest count across all species in a tree were best predicted by each of tree basal area, crown dieback-weighted basal area, total dead stem count or total dead wood volume. The four predictor variables were highly correlated (correlation coefficient > 0.62) so we separately modelled each predictor for both species richness and total nest count (eight total models) and evaluated model fit by comparing Akaike Information Criterion (AIC) values (electronic supplementary material, table S1).

Individual ant species could also respond differently to tree characteristics and to the presence of other ant species on a tree. To explore this, we created four separate linear models each of eight different response variables: a binary presence/absence variable for each of the four focal ant species (four response variables) or the total nest count in a tree for each species (four response variables). All models included four predictors; three variables reporting total nest counts for the non-focal species of the analysis along with one of the four tree characteristic metrics listed above (32 models in total). For the presence/absence tests, we used generalized linear models with binomial errors and a log link function [68]. We reduced the models using backwards stepwise AIC selection [69] and AIC comparisons were used to determine best-fit model. Final models for all 32 tests are provided in electronic supplementary material, table S2.

#### (ii) Nest network analyses

We explored how the network of all ant nests within a tree was shaped by tree-level characteristics and the composition of the local ant community. We constructed six linear models with one of two response variables: the average physical distance or the average junction distance between any two nests in a tree. All models included five predictors; four predictors representing number of nests occupied by each of the four focal species, and a fifth predictor related to one of three metrics of dead wood availability within each tree (weighted basal area, dead twig count or total dead wood volume). To explore how the nest networks of each focal species responded to tree characteristics and the presence of other ant species, we evaluated another 24 models with the same set of predictors, with the response variables as either the average physical distance or the junction distance among nests of the same species in each tree (intraspecific nest distances). We reduced the models using backwards stepwise AIC selection and AIC comparisons were used to determine best-fit model. The final models for each of the tests are provided in electronic supplementary material, table S3.

Individual ant species could also display species-specific spatial nesting patterns. To examine these patterns, we explored differences in pairwise distance between all focal nests within each tree. We expanded the initial dataset by adding 19 more trees (*n* = 50 trees) with complete information on the ant community and nest networks, but lacking complete dead wood data. We documented 365 intraspecific pairwise nest distances split among the four focal ant species (e.g. distance between two *C. varians* nests, two *P. ejectus* nests, two *P. gracilis* nests or two *P. simplex* nests). We used two linear mixed models with physical or junction distance between two nests as the response variable, nest pair category as the predictor variable (four levels; one for each species) and Tree ID as a random grouping factor. We used a Tukey's *post hoc* test to explore differences in means per category.

#### (iii) Individual nest analyses

We first explored whether the mean volume of occupied stems differed among the four focal species and from the mean volume of unoccupied dead stems on a tree. We chose volume because nest quality is generally determined by cavity volume as it dictates how much space there is for colony growth (e.g. [39]). We fit stem volume as a function of species nest occupancy using a mixed-effects ANOVA, where species nest occupancy was a categorical variable with five levels (unoccupied, *C. varians*, *P. ejectus*, *P. gracilis* and *P. simplex*). Tree ID was included as a random grouping factor, and we used Tukey's *post hoc* tests to explore any differences among ant species nest selection.

We next explored whether the contents of a nest were predicted by stem volume, nesting ant species identity, and distance to the nearest nest of each of the four focal ant species (eight predictors; two for each species to account for two different distance measurements) using zero-inflated generalized linear mixed models with negative binomial errors and a log link function. [41]. In each model, we included either the total count of the combination of all adult ants and brood, only adult ants or only brood as the response variable. We selected these metrics as nest defensibility is determined by defensive strategies of individual ant species [38,40,41,51] and competitor pressure depends on the neighbourhood of enemies trying to usurp the nest for themselves [41]. Ants will also differentially move their brood and redeploy adult ants based on perceived threat or nest defensibility. We started with 24 models (three response variables with all models including stem volume, nest ant species identity and one of eight distance measures). Model reduction and AIC comparison resulted in all models reducing to only ant species identity and the interaction between species identity and distance to the nearest *P. gracilis* nest as the best-fit models. Tree ID was treated as a random grouping variable for all models. We used a Tukey's *post hoc* test to explore any pairwise differences in nest contents between ant species.

All statistical tests were performed in the R environment version 4.2.2 [70] including packages *lme4* [71], *lmerTest* [72] and *glmmTMB* [73]. In all models, metrics of tree size, species richness, network distances, stem volumes and individual ant counts were log transformed to meet model assumptions where necessary and to match the expectation of a log–log linear relationship between species richness and area measurements [74]. Finally, we confirmed normality for all parametric models using Shapiro–Wilk tests on model residuals and performed residual diagnostics to confirm models conformed to model assumption using *DHARMa* [75].

## 3. Results

### (a) Resource patch scale: whole tree

**Figure 1.**
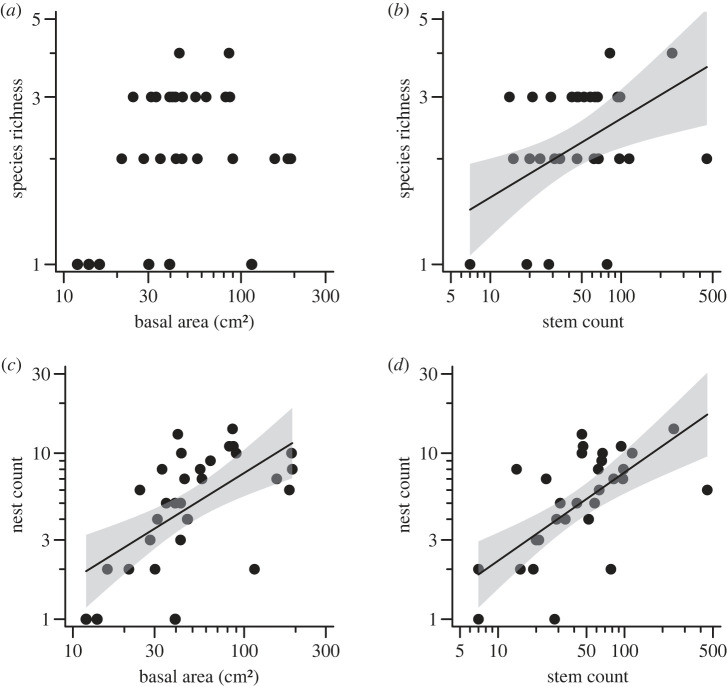
The relationships between arboreal ant species richness and total nests across tree basal area (*a,c*) or the total count of dead stems in a tree (*b,d*). Regression lines with 95% CI (shaded region) are included when there is a significant relationship (electronic supplementary material, table S1). Axes are on log-scales.

At the scale of discrete resource patches, whole trees with more nesting habitat had more ant nests and ant species, but species richness was not related to tree size. Specifically, trees with larger basal areas and more dead stems had more ant nests, but only dead stem count predicted variation in ant species richness. Basal area alone did not predict the number of ant species (figure 1; electronic supplementary material, table S1). Of the three metrics of dead wood availability, the number of dead stems in a tree was the best predictor of both species richness and total nests (electronic supplementary material, table S1).

The four focal ant species responded differently to nesting resource availability and potential species interactions. *Cephalotes varians* was influenced only by nesting resource availability, with its likelihood of being present on a tree higher in trees with more dead wood, with weighted basal area specifically functioning as the best predictor (electronic supplementary material, figure S2 and table S2). By contrast, the two native small-bodied *Pseudomyrmex*, *P. ejectus* and *P. simplex*, were influenced only by the presence of other ant species. These two species generally did not co-occur, but when both were present in a tree, the number of nests of the two species were negatively associated (electronic supplementary material, figure S3). Additionally, *P. ejectus* frequently co-occurred with *C. varians*, whereas *P. simplex* had a lower frequency of occurrence in trees that also hosted the non-native *P. gracilis* (electronic supplementary material, figure S4*a,* S4*b* and table S2). *P. gracilis* was influenced only by resource availability, establishing more nests in trees with a higher volume of dead wood (electronic supplementary material, figure S5 and table S2).

### (b) Resource network scale: nest network

**Figure 2.**
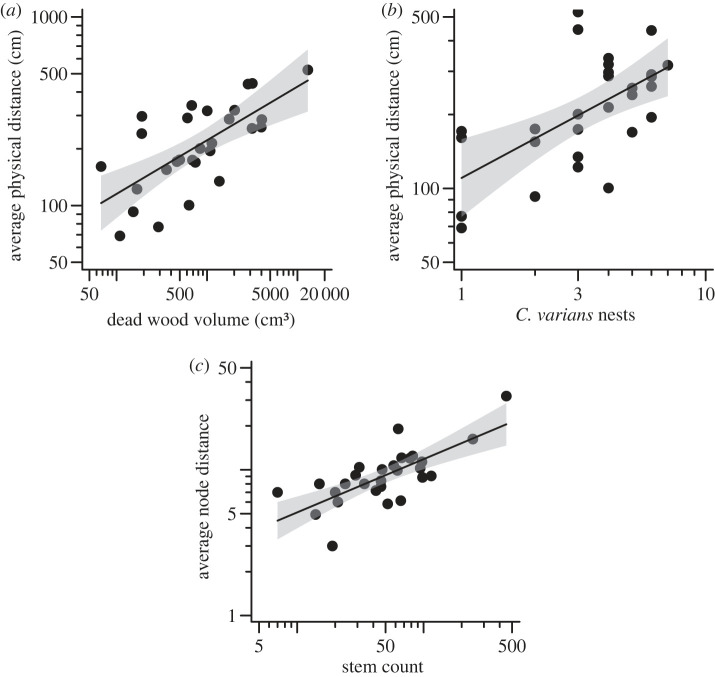
Statistically significant relationships between the community-wide distances between nests and the amount of dead wood or the number of ant nests in a tree. The shaded region around the regression lines indicates the 95% CI. Axes are on log-scales.

For the resource network scale, both habitat availability and the presence of specific ant species shaped the community-wide nest network formed by all resident colonies on a tree. Specifically, both the physical distance and junction distance between any two nests in a tree increased with increasing dead wood availability, with dead stem volume acting as the best predictor for physical distance (figure 2*a*) and dead stem count as the best predictor for junction distance (figure 2*b*; electronic supplementary material, table S3). In addition, the average physical distances between ant nests in a tree were higher in trees with more *Cephalotes* nests (figure 2*c*).

**Figure 3.**
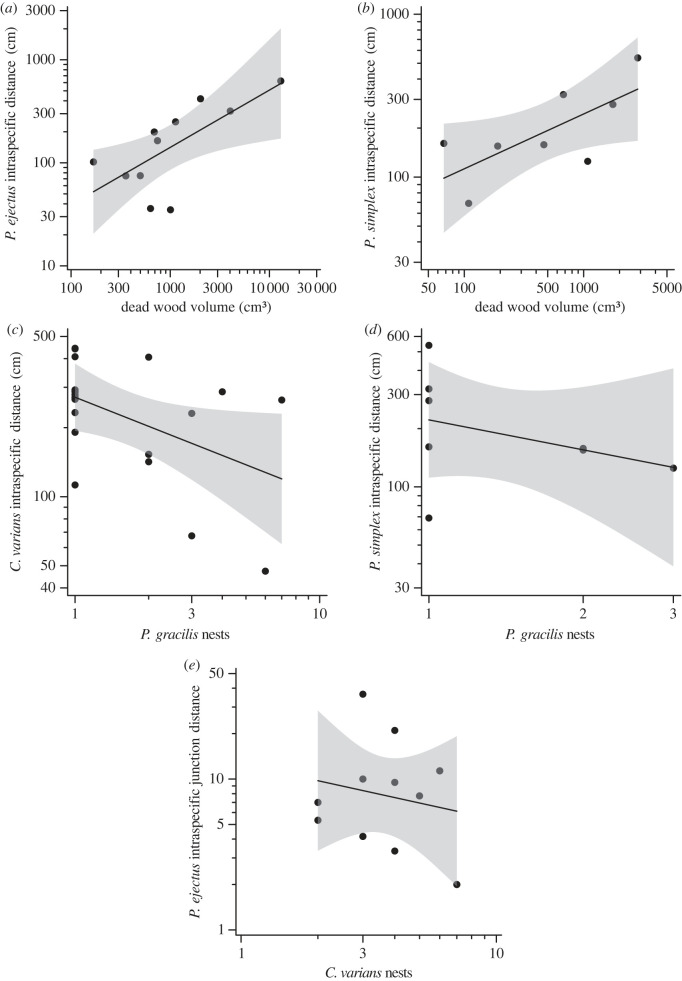
Statistically significant relationships between intraspecific nest distances (physical or junction distance) for different focal ant species and dead wood availability or the number of nests of other ant species in a tree. The shaded region around the regression lines indicates the 95% CI. Distance and volume measures are on a log-scale.

The intraspecific nest networks of individual ant species responded differently to resource versus competitor pressures. The nest networks of *P. ejectus* and *P. simplex* were more spread out in trees with greater dead wood volume (both physical and junction distances for *P. ejectus* and physical distances for *P. simplex*; figure 3*a,b*; electronic supplementary material, table S3). *Cephalotes* and the two native *Pseudomyrmex* species also responded to competitor abundance. Specifically, the average intraspecific nest distances for both *C. varians* and *P. simplex* was smaller in trees with greater numbers of *P. gracilis* nests (figure 3*c,d*; electronic supplementary material, table S3). *Pseudomyrmex ejectus* also had a more clustered nest network in trees with more *C. varians* nests (figure 3*e*; electronic supplementary material, table S3), whereas *P. simplex* had a less clustered nest network in trees with *C. varians*. Neither resource variables nor other ant species influenced the intraspecific distances among *P. gracilis* nests.

Measuring all intraspecific pairwise nest distances also revealed species-specific nesting patterns. *Cephalotes varians* and *P. ejectus* nests were, on average, more closely clustered together in space (both physical and junction distances) than the nests of *P. gracilis* or *P. simplex* (electronic supplementary material, figure S6; global tests—*F*_3,355_ > 7.62, *p* < 0.0006; Tukey Honestly Significant Difference (HSD)—*z* > 2.67, *p* < 0.04).

### (c) Individual resource scale: individual nests

**Figure 4.**
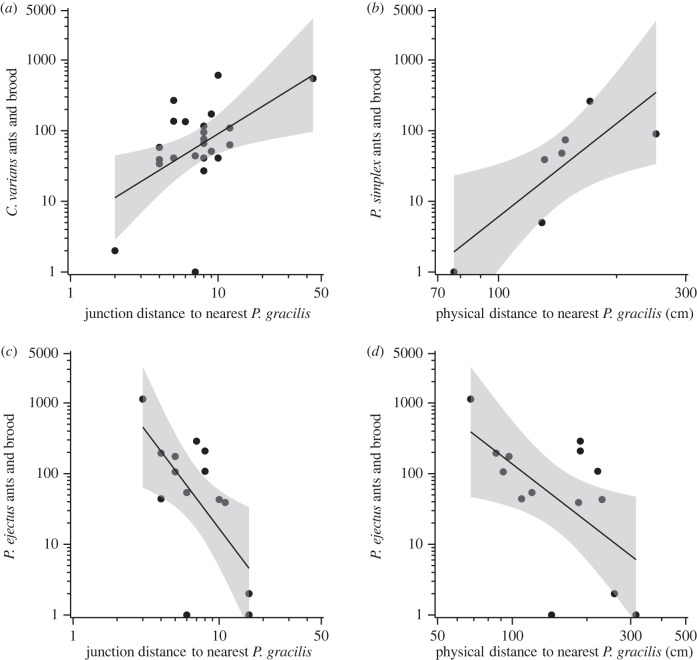
Statistically significant relationships between the contents of ant nests versus the distance to the nearest nest of *P. gracilis*. The shaded region around the regression lines indicates the 95% CI. Axes are on log-scale.

At the scale of an individual resource, defined by an individual ant nest within a nest network, *C. varians*, *P. ejectus* and *P. gracilis* all nested in stems that were of similar size and were larger than the average unoccupied dead stem on a tree (electronic supplementary material, figure S7). By contrast, *P. simplex* nested in smaller stems that were similar to average size of unoccupied dead stems on a tree (electronic supplementary material, figure S7; Tukey HSD—*z* > 2.94, *p* < 0.02). Exploring nest contents revealed that *C. varians* had more adult ants per nest than *P. ejectus* or *P. gracilis* (electronic supplementary material, figure S8; Tukey HSD—*z* > 3.01, *p* < 0.02). In addition, *C. varians* and the two native *Pseudomyrmex* ants showed consistent patterns for how they distributed brood and adult ants relative to their proximity to a *P. gracilis* nest (electronic supplementary material, table S4). Specifically, *C. varians* had fewer total ants and brood in nests that were only a few junctions from the nearest *P. gracilis* nest (figure 4*a*), and *P. simplex* had fewer total ants and brood in nests that were physically closer to *P. gracilis* nests (figure 4*b*). By contrast, *P. ejectus* had more ants and brood in nests closer to nests of *P. gracilis* measured by both physical and junction distance (figure 4*c,d*).

## 4. Discussion

Our results broadly support our central hypothesis that resource availability has an overarching influence on local community structure, and that species interactions emerge as more important at finer-grained local scales. Specifically, we observed that availability of dead wood was the primary driver of ant diversity at the scale of a tree, that the nest network was shaped by interactions between resources and competition, and that the distribution of ants within a nest was entirely driven by competition. Resource limitations and competitive species interactions are frequently proposed as contrasting drivers of diversity in arboreal ant communities [40,61,76,77]. By incorporating multiple spatial scales into this current study, we are able to demonstrate that both processes are acting in tandem and that the strength of their effects is scale dependent. These findings provide rare empirical support for the theoretical and computational framework of habitat characteristics imposing limitations on local diversity prior to [78,79] or in concert with [80,81] species interactions. The results also further highlight the need to match observations to the scale at which interactions occur, to avoid masking competition and other biotic interactions [13].

Species–area relationships are common among taxa and across spatial scales [82], but it is typically unclear what specific resources are underpinning these relationships [83]. At the scale of a whole tree, representing a discrete resource patch, we did not detect a direct relationship between species richness and tree size (basal area) despite this relationship being a common feature of arboreal ant communities [30–32,45]. We instead detected a species–resource relationship between ant species richness and dead wood availability, suggesting that nest site availability is the specific habitat limitation underlying area-based relationships within this arboreal ant community. We expect that this trend is widespread among arboreal ant communities, and we predict that where species–area relationships exist between tree size and ant species richness, incorporating measurements of nesting resources would better predict diversity patterns. Ultimately, habitable patch area is a proxy for a broad series of scale-dependent resources and ecological processes ranging from likelihood of encounter during dispersal, available food and nest sites and proximity to competitors [11,84]. These patterns suggest that habitat limitations on a community can be masked when fine-scale resource availability is not considered [13].

Resource availability provides a foundation for determining local diversity and community structure [14,18] but species interactions and behaviours can mediate the final outcome and dynamics [1,3,21]. We demonstrate that the arboreal ants in this forest follow these general trends in terms of their resource networks, represented by networked nests that each colony occupies. For example, *C. varians* is a nest defence specialist that typically clusters its nests in a laboratory setting [25] and has a soldier caste that uses an armoured head dish to barricade the colony's nest entrances [59]. In a natural setting, we demonstrate that, compared with commonly co-occurring species, *C. varians* not only has the most clustered intraspecific nest network, as would be indicative of a species prioritizing defensibility, but also further shrinks its network in the presence of the aggressive, non-native competitor *P. gracilis*. By contrast, *P. gracilis* disperses its nests broadly across a tree crown, as expected of a non-native under less competitive pressure [3,85,86]. In addition, *P. simplex* has a broadly dispersed nest network but has a significantly contracted nest network in the presence of *P. gracilis*. Collectively, these observations lend additional support to the idea that *P. gracilis* is using its widely dispersed nesting strategy to limit nest acquisition by other members of the community and that less aggressive native species shrink their nest networks in response to this competitor.

Biotic interactions at finer spatial scales can also have meaningful impact on species growth within the community, even when it is not reflective in measurements of species richness or composition. However, the subtle impact of species interactions on growth patterns are frequently impossible to detect without extensive multi-year studies tracking individuals through time [87]. Here, we were able to collect data across local spatial scales, including at the fine-grained local scale of individual resources via distances between nests and the distribution of ants among nests. These data allowed us to demonstrate in a snapshot that the aggressive, non-native *P. gracilis* exerts competitive pressure on *C. varians* and *P. simplex* that limits the spatial extent and pattern of colony growth. More specifically, both ants have more clustered networks in trees with *P. gracilis* and tend to have fewer ants and brood in nests near *P. gracilis*. By contrast, *P. ejectus* tends to have more ants and brood in nests nearest to *P. gracilis*. Considering *P. ejectus* and *P. simplex* exhibit almost complete competitive exclusion, the distribution of *P. ejectus* ants in nests near to *P. gracilis* could arise from a form of competitive release [88,89] wherein *P. gracilis* limits *P. simplex* allowing for *P. ejectus* to better perform nearer to *P. gracilis*. Experimental manipulations of the ant community would be necessary to confirm these observations, but being able to detect these patterns further highlights the value of multi-scale collection regimens for local community ecology data.

The outcome of multispecies interactions on the diversity and stability of ecological communities is notoriously difficult to understand and predict [6,22,90]. The majority of work on the subject is carried out in laboratory or mesocosm experiments [19,91], in plant systems where individuals can be more easily tracked [92,93] or via simulations [94]. The arboreal ant community of the Florida Keys hammock forest exhibits considerable utility in parsing the outcome of multispecies interactions in a complex but manageable animal community of conservation concern. The results of this study suggest that while nest site availability is the main determinant of ant species richness and abundance at the scale of a tree, competitive interactions between species shape the spatial distribution of nests within trees and ants within nests. Experiments modifying nest site availability via artificial nest additions [32,33,40,61] and modifying community structure via relocating ants species among trees [35] could provide further evidence for the outcomes recorded here. The arboreal ant community of the Florida Keys presents an opportunity to explore ecological processes across multiple scales of ecological organization in a system that is both accessible and amenable to experimental manipulations. Ultimately, the key to determining the drivers of diversity is matching observations to the scale where interactions occur.

## Data Availability

All data included in this project are available via Dryad Digital Repository: https://doi.org/10.5061/dryad.h70rxwdpr [95]. Supplementary material is available online [96].
